# Patient involvement in preparing health research peer-reviewed publications or results summaries: a systematic review and evidence-based recommendations

**DOI:** 10.1186/s40900-020-00190-w

**Published:** 2020-06-24

**Authors:** Lauri Arnstein, Anne Clare Wadsworth, Beverley Anne Yamamoto, Richard Stephens, Kawaldip Sehmi, Rachel Jones, Arabella Sargent, Thomas Gegeny, Karen L. Woolley

**Affiliations:** 1 Envision the Patient – Envision Pharma Group, Suite 5.11, 5th Floor, 1 Lyric Square, London, W6 0NB UK; 2 Alligent EU – Envision Pharma Group, Wilmslow, UK; 3 Osaka University, Osaka, Japan; 4 Hereditary Angioedema Japan (Registered NPO), Hyogo, Japan; 5 Hereditary Angioedema International (Registered NPO/Charity), Fairfax City, VA USA; 6 Consumer Forum, National Cancer Research Institute, London, UK; 7 Research Involvement and Engagement, London, UK; 8 International Alliance of Patients’ Organizations, London, UK; 9 Swii.ch Health, Manchester, UK; 10 Curo – Envision Pharma Group, London, UK; 11 Engage – Envision Pharma Group, Southport, CT USA; 12 ProScribe KK – Envision Pharma Group, Tokyo, Japan; 13 University of Queensland, Brisbane, Queensland Australia; 14 University of the Sunshine Coast, Maroochydore DC, Queensland Australia

**Keywords:** Patient and public involvement, Patient participation, PPI, Research reporting, Systematic review, Authorship, Medical writing, Clinical trials, Health research, Patient author

## Abstract

**Background:**

There are increasing calls for patient involvement in sharing health research results, but no evidence-based recommendations to guide such involvement. Our objectives were to: (1) conduct a systematic review of the evidence on patient involvement in results sharing, (2) propose evidence-based recommendations to help maximize benefits and minimize risks of such involvement and (3) conduct this project with patient authors.

**Methods:**

To avoid research waste, we verified that no systematic reviews were registered or published on this topic. We co-created, with patients, a PRISMA-P–compliant protocol. We included peer-reviewed publications reporting the effects of patient involvement in preparing peer-reviewed publications or results summaries from health research studies. We searched (9/10/2017) MEDLINE, EMBASE and the Cochrane Database of Systematic Reviews, and secondary information sources (until 11/06/2018). We assessed the risk of bias in eligible publications and extracted data using standardized processes. To evaluate patient involvement in this project, we co-created a Patient Authorship Experience Tool.

**Results:**

All nine eligible publications reported on patient involvement in preparing publications; none on preparing results summaries. Evidence quality was moderate. A qualitative synthesis of evidence indicated the benefits of patient involvement may outweigh the risks. We have proposed 21 evidence-based recommendations to help maximize the benefits and minimize the risks when involving patients as authors of peer-reviewed publications. The recommendations focus on practical actions patient and non-patient authors can take before (10 recommendations), during (7 recommendations) and after (4 recommendations) manuscript development. Using the Patient Authorship Experience Tool, both patient and non-patient authors rated their experience highly.

**Conclusions:**

Based on a systematic review, we have proposed 21 evidence-based recommendations to help maximize the benefits and minimize the risks of involving patients as authors of peer-reviewed publications.

## Plain language summary

- This research project is a systematic literature review. This is a structured way to look at all the published research on a topic.
- This review looked at patient involvement in sharing results from health research studies, including clinical trials. Results from health research studies are shared in different ways, such as:
  - Being published in a scientific journal (called a peer-reviewed publication);
  - Being presented in a lay summary (called a clinical trial results summary).
- The researchers wanted to know:
  - What are the benefits and risks of involving patients in sharing results?
  - What recommendations could help to increase the possible benefits and reduce the possible risks?
  - What was the experience of patient authors taking part in this review project?
- The researchers searched for published research from different sources. They found 167 articles, of which nine were relevant.
- The researchers found that:
  - All nine articles were about patient involvement in peer-reviewed publications. None of the articles were about patient involvement in clinical trial results summaries.
  - The benefits of patient involvement in sharing results appeared to outweigh the risks.
- Based on the evidence they found, the researchers made 21 recommendations.
  - These could help increase the possible benefits and reduce the possible risks when involving patients as authors of peer-reviewed publications.
- Overall, the patient and non-patient authors involved in this review project had a good experience.
- These results suggest that patients should be involved in sharing research results. There are now recommendations on how to involve patients as authors on publications.

See Additional file 1 for an infographic plain language summary.

## Background

The importance of ethical and substantial patient involvement in medicines development is being acknowledged by stakeholders [1, 2]. Patients are becoming informed, empowered and active partners in their healthcare, and are calling for increased involvement across the entire medicines development life cycle [3, 4]. In response, stakeholders are recognizing the need for internal process and cultural change. Regulators, including the European Medicines Agency and the US Food and Drug Administration, have declared patient involvement to be a priority, and payers are increasingly taking the patient perspective into account in reimbursement decisions [1, 5]. The pharmaceutical industry is beginning to empower the patient voice in medicines development, partnering with international patient advocacy organizations and healthcare professionals to establish ethical frameworks and principles to facilitate patient involvement [6–8].

In conjunction with this patient-empowered evolution, patients are increasingly seeking health information from peer-reviewed publications, and this demand is likely to increase [9, 10]. Consistent with this interest from patients and their advocates, some medical journals are facilitating greater involvement of patients as authors, editors, peer reviewers and readers [11, 12]. In addition, increasing attention is being focused on involving patients in plain language summaries of publications of clinical trials and other types of research publications, building upon the European Medicine Agency’s regulatory requirement for lay summaries of clinical trial results [13].

However, in comparison to patient involvement in other phases of the health research life cycle, such as study design and recruitment, patient involvement in publications has lagged behind. Not only does the level of patient involvement in publications seem low, but the potential benefits and risks of patient involvement are not well understood. Such insights are needed to define evidence-based best practices, thereby enhancing the potential benefits and minimizing the potential risks of involving patients as publication partners (e.g. as authors or contributors).

Our objectives were to: (1) conduct a systematic review of the evidence on patient involvement in results sharing, (2) propose evidence-based recommendations to help maximize benefits and minimize risks of such involvement and (3) conduct this project with patient authors.

## Methods

### Protocol, registration and definition

This systematic review was registered in the PROSPERO database (PROSPERO 2018 CRD42018084452), conducted according to a prespecified protocol and reported in compliance with best-practice reporting guidelines for systematic reviews [14] and research involving patients [15]. For this study, we employed the PICO model to define the key elements in our research, which included peer-reviewed publications that have investigated the effects of having patients (P – Participants) engaged as authors of or contributors to (I – Intervention) peer-reviewed publications. We quantified the number of these publications (Outcomes) and compared this with the number of publications on patient involvement in the preparation of lay summaries of clinical trial results (Comparator).

To minimize the risk of research waste, we searched (5 June 2017) the PROSPERO database to ensure we were not duplicating a planned or ongoing systematic review. We also registered our review on SYNAPSE, the Patient Focused Medicines Development (PFMD) repository for patient engagement initiatives (https://synapse.pfmd.org/initiatives/patient-involvement-in-preparing-clinical-research-peer-reviewed-publications-or-results-summaries-a-systematic-review).

Within this study, ‘patient’ was defined in broad terms, based on an existing definition [16, 17] and input from our patient partners. For this research, ‘patient’ refers to “people having or at risk of having medical condition(s), whether or not they currently receive medicines or vaccines to prevent or treat a disease” as well as “the family and those voluntarily caring for those with the medical condition(s), patient advocates and patient groups.”

### Eligibility criteria

We included peer-reviewed publications that studied the effects of patient involvement for sharing results of health research studies (all phases and all designs) either through a peer-reviewed publication or through a lay summary of clinical trial results (i.e. consistent with the regulatory requirements described in the EU Clinical Trials Regulation 536/2014 [Article 37] [13]). We considered publications, in any language, that were detected in searches of electronic bibliographic databases or secondary sources (see Information sources and search strategies section). We did not include unpublished data or abstract-only articles.

### Information sources and search strategies

We used primary and secondary information sources. For our primary source, we searched three electronic bibliographic databases (MEDLINE, EMBASE and Cochrane Database of Systematic Reviews) using the OVID database search interface. We used Medical Subject Headings (MeSH) and words/phrases related to patient involvement in health research peer-reviewed publications and lay summaries of clinical trial results. An experienced search strategist conducted the search of the three databases on 9 October 2017 (limits: 1 January 2015–9 October 2017; see Additional File 2 for full search strategies). References were exported to a reference management software program (EndNote) and saved into a project-specific library within EndNote. Duplicate publications were removed.

We supplemented our primary information source with secondary information sources. These sources comprised the repository of patient involvement in research literature curated by the Patient-Centered Outcomes Research Institute (PCORI), PubMed’s ‘similar articles’ listings and the authors’ personal reference files. A search of the PCORI repository was conducted on 10 June 2018 via PCORI’s Engagement in Health Research Literature Explorer (limits: dissemination, 1 January 2017–10 June 2018); a search of the top 10 PubMed articles most related to eligible articles retrieved from our primary information sources was conducted on 11 June 2018; and authors’ personal references were scanned up until 30 May 2018.

### Study selection

We assessed publications for eligibility using a standardized process and a dedicated assessment team (three authors with predefined roles). One author (KW) selected potentially eligible articles based on a title and abstract screen, then two authors (LA, TG) independently selected eligible articles based on a review of the full text of each potential article. Differences in assessment were resolved by consensus among the assessment team.

### Data collection process and data items

Based on the protocol, we prepared a spreadsheet template to collect data from all eligible publications. The template was pilot tested and refined before use. Data were collected using a standardized process and a dedicated data collection team (three authors with predefined roles). Two authors (RJ, AS) independently extracted data from each publication and a third author (ACW) verified 100% of the data extracted for the primary outcome measure from every eligible publication, and verified 100% of the data extracted for the secondary outcome measures from a random sample of 25% of the eligible publications. Data (Additional File 3) were extracted for publication title, journal, year, authors, first author’s country, research sponsor, number of patient authors, patient author background, patient author recruitment process, author communication process, extent of patient involvement in preparing the publication or lay summary of clinical trial results, how patient involvement outcomes were assessed, benefits (to patient authors, other authors, the project), risks (to patient authors, other authors, the project) and best practice recommendations.

### Risk of bias in individual studies

We anticipated that there would be few randomized controlled trials that directly addressed our research question. As per the protocol, the Cochrane Collaboration tool for assessing bias risk (https://handbook-5-1.cochrane.org/chapter_8/table_8_5_a_the_cochrane_collaborations_tool_for_assessing.htm) was to be used to assess randomized controlled trials. The Newcastle-Ottawa Scale was used to evaluate the risk of bias for other study designs, although we recognize this instrument does have limitations (http://www.ohri.ca/programs/clinical_epidemiology/oxford.asp).

### Summary measures

Our primary outcome measure was the number of peer-reviewed publications that investigated the effect of patient involvement on preparing peer-reviewed publications.

Our secondary outcome measures were:

- The number of peer-reviewed publications that investigated the effect of patient involvement on preparing regulatory-standard lay summaries of clinical trial results;
- The quality of the evidence reported in the eligible publications;
- The number and the background (e.g. patient experts, clinical trial participants, patient advocacy group members) of patients contributing to the preparation of the publications or lay summaries of clinical trial results;
- The type of patient involvement (e.g. as authors, as non-author contributors);
- The number and type of patient involvement outcomes assessed (e.g. benefits, risks, best practice recommendations, other).

### Synthesis of results

As anticipated in the protocol, we did not synthesize data quantitatively due to heterogeneity in the eligible publications. We conducted a qualitative narrative synthesis of the data to describe patient involvement outcomes.

### Additional analyses

To assess the quality of the patient authorship experience in the present study, one author (KW) customized the original PFMD Patient Engagement Quality Guidance [16], which PFMD co-created with over 100 people from 51 organizations, representing patient associations, industry, academics, researchers, and external experts. The resulting draft Patient Authorship Experience (PAE) self-assessment tool was then updated with co-author feedback. The final PAE tool evaluates eight publication-relevant domains using a bipolar, five-point, psychometric, Likert scale (strongly disagree to strongly agree) [18]. The total score is determined by adding up the individual scores for each question and calculating a percentage out of a maximum score of 80. Two versions were created: one for patient authors and another for non-patient authors. All authors in this study completed the PAE tool.

To document the involvement of patients in this project, the GRIPP2 Short Form was completed [15].

## Results

### Quantity of evidence on patient involvement

**Fig. 1:**
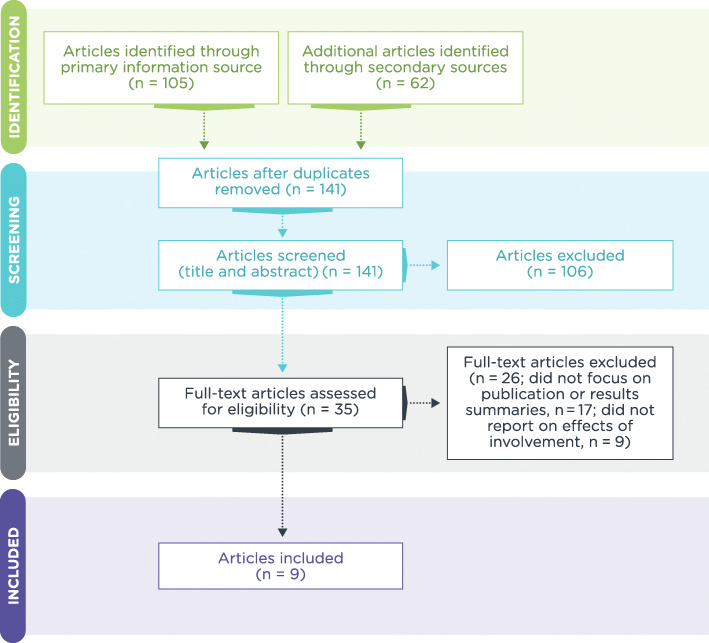
Flow of screened and eligible publications (PRISMA Flow Diagram)

The number of peer-reviewed publications that reported the effects of patient involvement on preparing peer-reviewed publications was low. An extensive search of primary and secondary information sources yielded only nine publications (Fig. 1). Of these nine publications, nine reported on the effects of patient involvement in preparing peer-reviewed publications; none reported on the effects of patient involvement in preparing regulatory-standard lay summaries of clinical trial results.

### Quality of evidence on patient involvement

The quality of evidence on patient involvement in preparing peer-reviewed publications was moderate. There were no published systematic reviews or randomized controlled trials focused on this topic. Assessments based on the Newcastle-Ottawa scale, indicated six of the nine studies were good quality and three were fair/poor quality (Table 1).

**Table 1:** Study characteristics and quality of the nine eligible publications

Ref	Study	Sponsor type	Patient involvement in publication (background)		Patient involvement in lay summaries of clinical trial results		Grading score (Newcastle-Ottawa Scale)	Therapeutic area	Country	Publisher
			Author	Contributor	Author	Contributor				
[19]	Absolom K, et al. Qual Life Res. 2015.	NIHR	Y (PPI group member)	Y (PPI group member)	N	N	Poor/fair quality	Oncology	UK	Springer Nature
[20]	Pollard K, et al. Int J Health Care Qual Assur. 2015.	Higher Education Innovation Fund, West of England University	N	Y (service user research partners)	N	N	Poor/fair quality	General	UK	Emerald
[21]	Hyde C, et al. Health Expect. 2017.	NIHR	N	Y (patient research user group members)	N	N	Good quality	Pain	UK	Wiley
[22]	Mann C, et al. Res Involv Engagem. 2018.	NIHR Health Services and Delivery Research Programme	Y (Patient Involvement in Primary Care Research group)	Y (Patient Involvement in Primary Care Research group)	N	N	Good quality	Chronic multiple morbidities	UK	BMC Springer Nature
[23]	Pérez Jolles MP, et al. Health Expect. 2017.	PCORI	Y (mentor parent group members)	Y (mentor parent group members)	N	N	Good quality	Mental health	US	Wiley
[24]	Howe A, et al. Res Involv Engagem. 2017.	NIHR Health Services and Delivery Research Programme	Y (PPI representatives)	Y (PPI representatives)	N	N	Good quality	General	UK	BMC Springer Nature
[25]	Forsythe L, et al. Qual Life Res. 2018.	PCORI	Y (patient advisor)	Y (patients, caregivers, advocacy groups)	N	N	Good quality	General	US	Springer Nature
[26]	Blackburn S, et al. Res Involv Engagem. 2018.	NIHR School for Primary Care Research	Y (PPI contributors)	Y (PPI contributors)	N	N	Good quality	General Primary care	UK	BMC Springer Nature
[27]	Barnfield S, et al. Res Involv Engagem. 2017.	Medical Research Council, UK	N	N	N	N	Fair quality	Stroke	UK	BMC Springer Nature

*NIHR* National Institute for Health Research, *PCORI* Patient-Centered Outcomes Research Institute, *PPI* patient and public involvement

### Type of patients involved and type of involvement

Patients were involved as authors in six of nine publications, and as contributors in eight of nine publications; the majority of them were members of patient and public involvement (PPI) groups (Table 1).

### Evidence on benefits and risks of patient involvement

From the evidence reported, patient involvement was associated with benefits and risks, which we categorized into those that affected: (1) patients, (2) others (primarily researchers and clinicians) and (3) the actual research projects (Table 2). Based on the number of effects and the overall impact, the potential benefits of patient involvement appear to outweigh the potential risks. Key benefits include validation of research need, relevance and value; improved materials and processes; improved reporting and dissemination; enhanced credibility and trust; and development of new knowledge and skills. Risks focused on increased resource needs and opportunity costs; navigation of power dynamics; and risk of nonrepresentative insights from less diverse PPI groups.

**Table 2:** Reported benefits and risks of involving patients in preparing peer-reviewed publications

Reference #	Benefits of patient involvement			Risks of patient involvement		
	For patients	For others	For the project	For patients	For others	For the project
[19] Absolom et al.	Feel that contribution is valued and actioned; gain new skills and knowledge of the research process; opportunity to use transferrable skills; increased confidence as a patient representative	Enrich researcher understanding of the topic; learn how to tailor projects around patients’ availability, health status and interest	Patients raised new issues, such as sensitive data management and cultural diversity; patients asked questions that produced useful discussion	May initially feel daunted by experienced researchers and clinicians; time needed to get to grips with complex information	Time investment to brief patients before meetings	Patient withdrawal due to ill health can produce team gaps and make it challenging to sustain a volunteer-run committee in the longer term
[20] Pollard et al.	Improved confidence and skills, including facilitation, handwriting and speaking; feel supported by having an academic researcher dedicated to evaluating the patient experience	Gain insight that early planning and consistent guideline use around enhancing patient involvement are important; gain greater awareness of challenges such as meetings and events timing, accessibility and communication	Stakeholder diversity in meetings (academics, practitioners and patients)	Physical challenges of involvement, such as fatigue, difficulty speaking and difficulty writing; patients may not feel confident, or may have difficulty capturing the group’s attention; logistical challenges, such organizing transport to and from meetings	Other stakeholders may be cautious of patient involvement	Need to structure projects to address patients’ needs (e.g. fatigue, mobility and communication concerns)
[21] Hyde et al.	Opportunity to input into study design, results reporting and dissemination plan; ensure that patient priorities are directly reflected in the research; patients plan their own roles including content review, attending and giving presentations and contributing their perspective at conferences; involvement is valued and non-tokenistic	Support in identifying new research topics; reassurance that patients identified similar ideas (validity of input) and new ideas (innovative input) to researchers; develop strategies to help manage patient involvement challenges and establish best practice	Help ensure that the project is funded; refinement of the project scope by identifying gaps in the published literature; results interpretation and the dissemination plan more clearly reflect patient priorities	Navigation of time pressures and power dynamics; no evaluation of the experience of the patients themselves	Navigation of time pressures and power dynamics	Best practice recommendations not validated by assessment of patient experiences; patient insights may not be fully representative, as the patient group was non-diverse and carers and stakeholder organizations were not involved; potential for introducing research bias
[22] Mann et al.	Enjoy being part of a group; gain confidence and freedom to challenge researchers; learn listening skills and how to share views; training opportunities; sense of ownership towards the trial; feel listened to and valued; sense of making a difference	Gain insights from beyond the academic world, with patients as a ‘sounding board’ and ‘reality check’; improve training course for trial clinicians following patient input; increase knowledge of patient groups and organizations; learn communication skills; sense of the research being worthwhile and rewarding; more favourable perception of the potential of patient involvement; success in overcoming tokenism	Improve documents and webpage used in the trial, including better accessibility; gain advice on data collection and analysis methods; input into dissemination plan and initiation of publication; creation of an environment for substantial discussion and challenge	Conflict over differing participation requirements between patients; feel ‘taken for granted’ and perspectives not valued; communication challenges posed by patient group diversity; difficulty attending meetings due to illness, treatment, employment or other commitments; frustration at limited input into study design; non-concordance between patient and researcher priorities	Defensiveness and lack of respect for patient perspectives; frustration at lack of patients’ understanding of the constraints of the research, and at not being able to incorporate all of their good ideas; non-concordance between patient and researcher priorities	Patient insights may not be fully representative, as group had high literacy levels compared with the general population
[23] Pérez Jolles et al.	Sense of motivation and satisfaction at helping other patients and carers; meetings planned to fit around patients’ schedules; meetings conducted in preferred language; remuneration for participation	Gain insights on language to use to build trust with patients	Recommendations to improve study recruitment and retention; validation of and improvements to primary outcome measure and study tool; validation of analytical approach; credible, patient-led presentation of results to broader community; co-creation of visuals to enhance research presentations and publication; recommendations for further research	Challenging for patients to fully participate in results dissemination, e.g. unable to travel to a national conference to present due to competing responsibilities	None reported	Patient insights may not be fully representative, as group came from one community
[24] Howe et al.	Opportunity for training around research and providing insights; growing confidence in role over time; gain new, rewarding skills; remuneration for participation; feel supported and valued	Growing confidence in role over time; help ensure accountability to funders and the public; successful relationship-building	Shaping of the research process, including study documents and analysis methods; collection of new data; improved communication through anticipating accessibility challenges; input into dissemination plan; development of a tool for reflective practice	Tensions between patient members on the relative value of different roles (co-researcher vs. advisory committee); feeling ‘on the periphery’ in certain roles; feel ‘lost’ through jargon, procedures and lack of support; accessibility challenges (literacy, mobility, distance, digital); remuneration may affect taxes and benefits	Time cost, i.e. completing reflective practice, explaining jargon, longer meetings and providing help with technical aspects of research	Patient insights may not be fully representative, as the group was not gender-balanced; risk for team relationships to become obligated, collusive or dependent; risk of personal needs or priorities influencing patient input
[25] Forsythe et al.	Satisfaction from presenting and generating interest in the research	Improved skills to communicate with patients	Enhanced study recruitment and retention; validation and innovation of research topics, interventions, outcomes and measures; adapt materials and interventions to be more culturally or linguistically appropriate; modify intervention to be less burdensome for patients; contribution to data collection; new ways to share results; new audiences to reach and improved communication with different audiences; increasing credibility of study findings	Challenges with scheduling and logistics; limits on engagement due to health problems	Challenges with scheduling and logistics; difficulty identifying and fully involving diverse partners	Potential impact of challenges, such as managing different perspectives, on the way the project team works together
[26] Blackburn et al.	Increased knowledge of own condition, treatment options and how to access services; gain skills, opportunity for formal training; understanding of research and research processes; positive emotional impact of meeting new people and feeling of contribution; payment for some activities	Gain better understanding of a condition and insights on lived experience; increased motivation through the enthusiasm that patient contributors bring; increased impact of research; raise profile of institution and patient involvement centre of excellence; ensure resources are channeled into important topics; guidance on presenting results to non-researchers	Improved relevance, clarity and accessibility of materials, surveys and processes; setting and maintaining focus on the research question; maintain realism; increased recruitment and follow-up rates; validation of project and findings; ensure research is beneficial to the patient group; support with data interpretation; promotion of outputs; generate new or future research questions	Challenging to fully participate due to changes in health status, availability and other commitments; financial costs may not be reimbursed; potential impact on benefits; opportunity cost for other activities such as paid work or childcare	Time costs, such as recruitment, meetings and communication; project management challenges, i.e. if patient contributors are unreliable; opportunity cost for research time due to diversion of funds to patient involvement; increased pressure and stress; sensitivity to criticism	Patient insights may not be fully representative as groups are homogeneous (i.e. if patients encourage friends to participate); risk of duplicating efforts, i.e. patient involvement and qualitative work; patient contributors may be unreliable; financial costs such as travel, meeting and venue costs, IT and other infrastructure, and payment for patient contributors to attend conferences
[27] Barnfield et al.	None reported	Avoid ‘cherry picking’ perception through patient selection of relevant PLS for distribution	Guide selection of patient-relevant PLS; improved content and layout of PLS to optimize readability and comprehension; identify jargon the research team may have missed; improvements to website for PLS distribution	None reported	Time and financial cost related to conducting focus groups	Patient insights may not be fully representative, as all patients had a high level of education and previous involvement experience; personal experience may introduce bias, i.e. preference for using emotive language

*PLS* plain language summary

### Evidence on best practices recommendations for patient involvement

Based on the evidence extracted from the eligible publications (Additional file 3), we developed a list of 21 recommendations that could help maximize the benefits and minimize the risks of involving patients in preparing peer-reviewed publications (Table 3). Each recommendation is linked to the eligible publications that provided the evidence to support that particular recommendation. To enhance practical use, the recommendations are categorized into actions that research teams could take before, during and after manuscript preparation.

**Table 3:** Evidence-based best practice recommendations for involving patients as authors

Stage of manuscript preparation	Recommendation	Reference # (evidence supports basis of recommendations)
Before	1. Ideally, involve patients in the question formulation stage (e.g. involve patients in publication planning to ensure publications address unmet needs that are relevant and important to patients)	[19, 21, 22, 24–27]
	2. Identify patient author candidates who are interested in contributing, have relevant expertise (e.g. lived experience) and can meet authorship criteria (i.e. no guest authorship); document consented contact details for patient authors in publication management software	[19, 23–25, 27]
	3. Clarify and document author and contributor roles and responsibilities (e.g. signed authorship agreements should help ensure expectations are clear and understood; patient involvement should be substantial; archive signed agreements)	[19, 21, 23–25, 27]
	4. Ensure support for patient authors from non-patient authors, especially the primary author and publication guarantor	[23, 25, 27]
	5. Appoint a designated contact person for patient authors to reach out to with queries (e.g. a Certified Medical Publication Professional who has publication expertise, project knowledge and time to support patient authors)	[23, 25, 27]
	6. Identify relevant publication and patient involvement guidelines that will be followed (e.g. CONSORT, GRIPP2, GPP3)	[24, 25]
	7. Check that funding facilitates patient author involvement (e.g. upfront payment of travel expenses for author meetings and conference presentations, translator fees if necessary)	[19, 21, 23–27]
	8. Prepare a publication timeline that facilitates patient author involvement (e.g. early delivery of materials to review; contingency time for unexpected unavailability – illness, employment, other commitments)	[21, 23–25, 27]
	9. Consider providing a publication induction guide and training for patient authors (e.g. plain language summary of GPP3, glossary of publication terms, overview of publication process)	[19–21, 23–25, 27]
	10. Consider how to proactively and systematically evaluate the effect of patient involvement (e.g. document feedback via publication management software; administer patient authorship experience tools)	[19, 21, 23, 25–27]
During	11. Recognize and respect diversity in the authorship team – everyone should contribute and be listened to. Patient authors can provide unique and useful input from their lived experience (e.g. they are not expected to be statisticians, clinicians, medical writers)	[19, 23, 25]
	12. Be flexible in how patient authors can provide input (e.g. telephone, email, in person)	[19, 22–27]
	13. Allow time before, during and after authorship meetings to address concerns and questions about patient authorship – from patient and non-patient authors	[24, 25, 27]
	14. Provide timely and regular feedback to patient authors on their contributions and group dynamics	[19, 21–23, 25–27]
	15. Consider presenting key results at authorship meetings and in publications that could make it quicker and easier for non-specialists to understand and interpret findings (e.g. use data visualization, flowcharts)	[20, 21, 26, 27]
	16. Recognize that patient authors may provide stronger contributions if able to provide input in their local language	[26]
	17. Document, in the manuscript, the involvement and role of patient authors (i.e. identify which authors are patients [e.g. Author Affiliation section] and describe their authorship contributions [e.g. Contributorship section])	[21, 27]
After	18. Provide updates on progress with the publication	[24, 27]
	19. Involve patients in the publication dissemination plan (e.g. raising awareness of the publication via patient advocacy groups, community and personal networks, social media platforms; contributing to and testing plain language summaries – ensuring cultural and linguistic appropriateness)	[19–23, 25–27]
	20. Encourage continued participation (e.g. patient authors presenting results – target geographically close conferences, leverage remote presentation tools; involvement in follow-up publication projects and publication steering committees)	[19, 22–24, 26]
	21. Consider preparing a companion publication on the effect of patient involvement	[19, 25]

*CONSORT* Consolidated Standards of Reporting Trials, *GPP3* Good Publication Practice 3, *GRIPP2* Guidance for Reporting Involvement of Patients and the Public

### PAE self-assessment tool

Overall, authors in this study rated their co-authorship experience highly. The average PAE tool score was 86% for patient authors (69 of 80; N = 3) and 90% for non-patient authors (72 of 80; N = 6). The authors identified areas to enhance future collaboration; for example, fewer assumptions on the part of the research team about patient authors’ prior experience and knowledge of publications and greater awareness of the extent of patients’ emotional investment in research involvement.

### Reporting of patient involvement in this project

Consistent with best-practice reporting for patient involvement in research [15], the involvement of patient co-authors in this project has been documented using the GRIPP2 Short Form (Table 4).

**Table 4:** Report of patient involvement in this systematic review and recommendations project (GRIPP2 Short Form)

Section and topic	Item
1: Aim	Report the aim of PPI in the study • To collaborate with patients as authors on a systematic review and, based on that review, propose evidence-based recommendations to help other authors (patient and non-patient authors) work together on publications. • To develop a tool, which could be shared with others, to help patient and non-patient authors evaluate their experiences of working together on publications.
2: Methods	Provide a clear description of the methods used for PPI in the study • Three patient partners were invited to join the research team at the study concept stage. They contributed to the development of the protocol for the systematic review, participated in author meetings, provided feedback on presentations (slide and poster presentations made to European and North American conferences) and manuscript drafts, co-created the plain language summary of the publication and contributed to the development of the Patient Authorship Experience tool.
3: Study results	Outcomes—Report the results of PPI in the study, including both positive and negative outcomes • Positive: The patient partners validated that this project would address an important and unmet need, which justified the initiation of the project. Their early and ongoing contributions provided ‘real world’ insights on the value of patient author involvement (e.g. they raised important points that non-patient authors did not). They provided candid comments and constructive criticism, both of which guided and strengthened the project, and they responded to requests for input, even under tight timelines and during holiday periods. Non-patient authors gained personal confidence and professional satisfaction that they were working on a project that would help other teams involve patient authors, ethically and effectively. They also obtained new knowledge and understanding about the complexities of academic publishing. • Negative: Additional time was required to develop new tools (e.g. plain language authorship guidelines, authorship agreements, Patient Authorship Experience tool) to ensure patient authors knew their rights and responsibilities, and to help ensure all team members could share their views and learn from this experience.
4: Discussion and conclusions	Outcomes—Comment on the extent to which PPI influenced the study overall. Describe positive and negative effects • Our patient partners had a critical influence on this project – if they did not see the need for it, then it would not have started. Their passionate belief that patients can and should be authors of publications, not ‘just’ contributors or readers, inspired the whole team. At the outset of the project, however, we had minimal guidance that was specifically relevant to working with patients as authors. We relied on general ‘PPI’ guidance documents, which were helpful, but not as specific as we would have liked.
5: Reflections/critical perspective	Comment critically on the study, reflecting on the things that went well and those that did not, so others can learn from this experience • One early issue research teams must address when wanting to involve patients is knowing which patients to invite and then establishing a culture that facilitates early and ongoing contributions. It is clear that if patients are to become authors, not all of them will already possess the skills set needed. For this project, we were in a fortunate position in that we knew patients who were keen to join the project early. Further, these patients were confident and passionate, and they understood that their expertise was valued. These elements contributed to a culture of openness, inclusiveness and efficiency. • There is recognition among our patient colleagues that if patients wish to become authors, then they will need to meet authorship criteria, that is, play a more substantial role than sense-checking or proofreading (important though these processes are). Inexperience with authorship may create grey areas early on for some patient (co-)authors. • On reflection, we did make some assumptions (e.g. logistical/technical assumptions about the ‘ease’ of joining WebEx calls; awareness of publication timelines/processes) that were unwarranted and we have learned how to address these. As we embarked on this project without the benefit of the 21 evidence-based recommendations that we have now proposed, we envisage that future projects will be enhanced by our ability to follow these recommendations.

*PPI* patient and public involvement, *GRIPP2* Guidance for Reporting Involvement of Patients and the Public

## Discussion

To our knowledge, this is the first systematic review of evidence on the effects of patient involvement in sharing results from health research studies. We identified nine publications that reported benefits and risks when patients were involved in sharing results through peer-reviewed publications. From this systematic review, we have proposed 21 evidence-based recommendations to help maximize the benefits and minimize the risks of involving patients as authors of peer-reviewed publications.

Consistent with the drive for greater involvement of patients in the development, regulation and reimbursement of medicines [1–4], there is growing recognition that patients should be more involved in sharing research results. However, in contrast to the proliferation of general guidelines on how to involve patients in the medicines life cycle [2, 3, 6, 7], there is limited guidance on how to involve patients in sharing results. Traditionally, results from all types of health research have been shared through peer-reviewed publications in medical journals. These publications have usually been authored by medical research scientists and clinicians. More recently, European regulatory requirements have prompted the development of lay summaries of results from certain types of clinical trial studies [13]. These regulatory-standard lay summaries are usually authored by research sponsors (e.g. pharmaceutical company staff or service providers). We reasoned that if patients are going to be more involved in sharing results through peer-reviewed publications or regulatory-standard lay summaries, then evidence-based guidance was needed to optimize patient involvement. This was the rationale for our systematic review.

From our systematic review, we found that evidence to guide patient involvement does exist, but it is limited in both quantity and quality. We identified publications that reported the benefits and risks of involving patients in preparing peer-reviewed publications, but we did not identify any publications that reported the effects of involving patients in preparing regulatory-standard lay summaries. Consequently, we have limited our evidence-based recommendations to how to involve patients as authors of peer-reviewed publications. As these appear to be the first set of evidence-based recommendations on this topic, we hope that developers of new or updated guidelines on how to prepare peer-reviewed publications will take our recommendations into account. For example, the Good Publication Practice guidelines (published in 2003 [28], 2009 [29] and 2015 [30]) are one of the most widely used guidelines for publishing industry-sponsored research [31], and are due to be updated. As many users of these guidelines will have limited or no experience with involving patients in publications, our evidence-based recommendations may help them maximize the benefits and minimize the risks of embracing this ‘innovation’ in publications. From our real-world experience of working with patient authors on this systematic review, it would have been helpful for our team (patient and non-patient authors) and our project if we had been able to follow these recommendations, before, during and after preparing this manuscript. We would also encourage guideline developers to address other areas of patient involvement in the publication ecosystem (e.g. inviting patients to serve on publication steering committees, co-creating plain language summaries of publications with patients, thanking patients for their participation in research studies, partnering with patients to raise awareness of research presentations and publications that are most relevant to patients).

From our systematic review, we also found that it was challenging to search the literature and to identify which authors may have been patient authors. Until these two issues can be addressed, research on this topic will not be as efficient or effective as it could be. For example, without the use of standardized keywords to interrogate databases (e.g. MEDLINE), we may have missed eligible publications. The lack of standardized keywords in an emerging area of research is not surprising, and we encourage stakeholders to propose and reach consensus on suitable keywords to enhance future literature searches. We suggest that even the adoption of ‘patient author’ would help research in this area. We did find that the yield of potentially eligible publications was higher from searching the PCORI’s Health Literature Explorer database, as some level of curation had already taken place. We encourage researchers with an interest in this topic to use the PCORI database (https://www.pcori.org/literature/engagement-literature). In terms of identifying which publications had patient authors, we found that there was no consistent or clear way that patient authors were described. We typically had to review the author affiliation, methods and acknowledgements sections of publications to detect which, if any, authors represented the patient perspective. Even if patient authors were identified, sometimes their listed affiliation was a university or hospital. A subsequent Internet search helped to clarify and confirm their role as a patient author (e.g. leadership role in a patient advocacy group, community advisory board). Understandably, these groups might be affiliated with a university or hospital and, quite rightly, this affiliation is given. However, it would help research in this area if such affiliations were complemented by a description of patient representative roles.

In addition to conducting this systematic review and proposing evidence-based recommendations, an important goal of this project was to partner with patients, as authors. We developed a number of tools, with patients, to help facilitate the involvement and evaluation of patient authors in publications and to help share our research findings with the patient community and other interested stakeholders. The PAE tool, with versions for patient authors and for non-patient authors, was a publication-specific adaption of the Patient Engagement Quality Guidance [7]. This guidance was developed by the not-for-profit organization, PFMD, with input from a large and diverse group of stakeholders [7]. We have made the PAE tool available for others to use for free ([18]; Additional Files 4 and 5). We welcome feedback on this tool, and hope that it helps patient and non-patient authors evaluate how well they worked together and where they might enhance their partnership. We have also developed, with patients and other stakeholders, a Plain Language Summaries of Publications Toolkit (https://www.envisionthepatient.com/plstoolkit/) [32]. We have also made this Toolkit available for others to use for free and welcome feedback on its use. The patient authors of this systematic review played a key role in using this Toolkit to co-create the plain language summary for this systematic review.

Although we have based our recommendations on a systematic review of the literature, we recognize that our research does have limitations and that additions to the literature over time will necessitate updates and expanded perspectives relevant to the expanding evidence. We found nine eligible publications and these all focused on the effects of patient involvement in preparing peer-reviewed publications, not regulatory-standard lay summaries. As more patients become involved in sharing results through publications or lay summaries, we encourage them and their non-patient partners to evaluate their experiences and publish their findings. We need to keep building the evidence base to guide best practice. We also encourage other researchers to expand and update the evidence base (e.g. by examining the grey literature; repeating our search as more papers on this topic are published).

We also acknowledge that while our recommendations are based on evidence, they need to be tested in ‘real-world’ situations. We have reflected on the recommendations and have recognized how applicable and valuable they would have been to this project, had they been available at the start. We have also started to apply these recommendations in new projects involving patient and non-patient authors, and the initial feedback from patient and non-patient authors has been positive. In terms of global use of these recommendations and the tools we developed, we welcome collaboration with individuals around the world who may be able to assist with translation and cultural adaptations.

## Conclusions

Published evidence on the effects of patient involvement in sharing results from health research studies is limited. From a systematic review of the literature, we have summarized the reported benefits and risks of involving patients in preparing peer-reviewed publications of health research studies. Based on this evidence, we have proposed 21 recommendations for involving patients before, during and after preparing a manuscript for publication. Understanding and learning from the experience of both patient and non-patient authors is equally important, and we piloted the PAE self-assessment tool as an initial step towards unlocking these insights. Researchers, patient partners and publication professionals may draw on these resources as they start or continue to collaborate on ethical, substantial patient involvement in publications.

## Supplementary information

**Additional file 1.** Infographic plain language summary.

**Additional file 2.** Summary of search strategies.

**Additional file 3.** Data extraction spreadsheet for nine publications included in this systematic literature review.

**Additional file 4.** PAE tool – patient author version.

**Additional file 5.** PAE tool – non-patient author version.

## Data Availability

Original articles are available through their respective publishers, some as open access.

## Abbreviations

PAE: Patient Authorship Experience
PCORI: the Patient-Centered Outcomes Research Institute
PFMD: Patient Focused Medicines Development
PPI: patient and public involvement
