# Supplementary material for: Patient involvement in preparing health research peer-reviewed publications or results summaries: a systematic review and evidence-based recommendations
Source: Res Involv Engagem. 2020 Jun 24;6:34. doi: 10.1186/s40900-020-00190-w (PMC7313171; doi:10.1186/s40900-020-00190-w)
Supplement: Supplementary file 1 — Additional file 1. Infographic plain language summary. [file 40900_2020_190_MOESM1_ESM.pdf]

This summary has been prepared using the Plain Language Summaries Toolkit,
co-created with patients (www.envisionthepatient.com/plstoolkit)

Should patients be involved in sharing results from health studies?

Date of summary: July 2019

The purpose of this plain language summary is to help you understand the findings from a recent research project.

- This summary reports the results from 1 project – a systematic literature review. A systematic literature review is a way to look at all the relevant published research on a topic.
- The results of this review may be different from the results of other reviews.

1 What did this research project look at?

- This research project was a systematic literature review. This means that researchers try to answer a research question by:
  1. Collecting all available and relevant published research
  2. Judging the quality of the evidence
  3. Giving the best answer they can, based on the evidence.

Systematic is pronounced < si-stuh-MA-tik>

- This review looked at patient involvement in sharing the results from health studies. Results from health studies are shared in different ways, such as:
  - Being published in a scientific journal (called a peer-reviewed publication)
  - Being presented in a summary written in plain language for people who took part in a clinical study (called a clinical trial results lay summary).
- Researchers and patients are interested in how to involve patients in sharing results. However, nobody had looked at all the published research on this topic to find out the best way to do that.
- For this research project, the research team included patient authors. The research team wanted to know:
  - What are the benefits and harms of involving patients in sharing results?
  - What recommendations could help to increase the possible benefits and reduce the possible risks?
  - What was the experience of patient authors taking part in this research project?

2 What evidence did the researchers include?

- The researchers searched for evidence from many different sources, between October 2017 and June 2018.
- They found 167 articles. Of these, 9 articles were relevant.

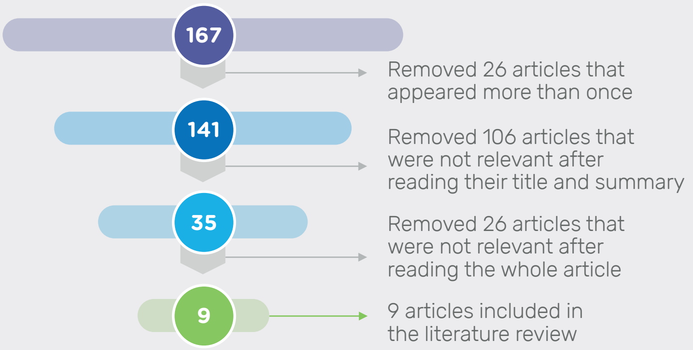

3 What were the results of this research project?

- All 9 articles were about patient involvement in peer-reviewed publications.
  - None of the articles were about patient involvement in clinical trial results lay summaries.
  - All articles were funded by government organizations.
- The quality of the evidence was moderate.
  - This means that the researchers have a medium level of confidence in the strength of the evidence. Some of the evidence came from studies that could have been better designed and reported.
- The benefits of patient involvement in sharing results appeared to outweigh the risks.

Risks

- Increase time and costs
- Lost opportunity to carry out other research
- Imbalance of power between researchers and patients
- Insights may not represent wider groups of patients

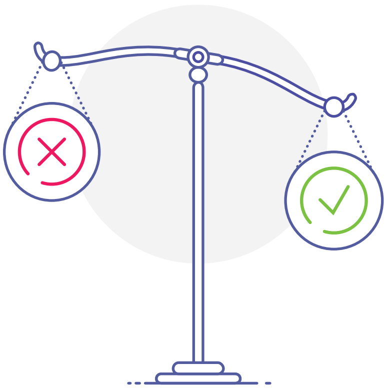

Benefits

- Easier to get funding for research
- Save time and costs
- Show that research is relevant and needed
- New and valuable input
- Improvements in the way research is done
- Improved sharing of results
- Patients and researchers learn new skills
- Improve trust in research results
- Identify new research topics

- Based on the evidence they found, the researchers made 21 recommendations.
  - These could help increase the possible benefits and reduce the possible risks when involving patients as authors of peer-reviewed publications.

Number of recommendations at each stage of preparing a publication

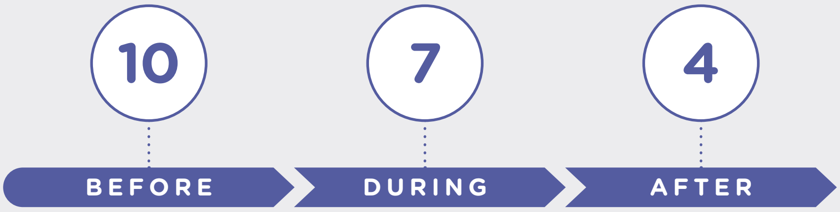

- Overall, the patient and non-patient authors involved in this research project had a good experience.

Average rating of author experience

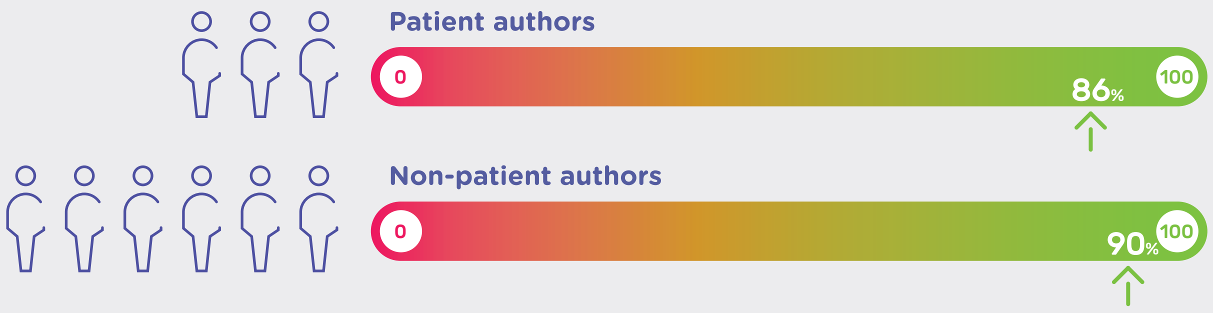

4 What were the main conclusions reported by the researchers?

- The evidence from this systematic review suggests that patients should be involved in sharing research results.
- When patients are involved in preparing publications, the benefits appear to outweigh the risks.
- There are now recommendations on how to involve patients as authors on publications. You can find these recommendations in the full article, which you can access through the link at the end of this summary.

5 Are there any plans for further projects?

This project is completed.

The researchers would like to study how well their recommendations work.

6 Who sponsored this study?

Envision Pharma Group provided limited financial support for administrative expenses.

The authors would like to thank all of the pioneers (patients and their research partners) who support patient involvement in medicines development. They have inspired our research.

Further information

- This study is listed on:
  - The PROSPERO database (#CRD42018084452; https://www.crd.york.ac.uk/prospero/display_record.php?RecordID=84452)
  - The Patient Focused Medicines Development Synapse platform (https://synapse.pfmd.org/initiatives/first-systematic-literature-review-planned-and-conducted-with-patient-experts-on-patient-involvement-in-preparing-clinical-trial)

QR CODE PLACEHOLDER

Scan this QR code to access a digital version of this summary

The full title of this article is: Patient involvement in preparing health research peer-reviewed publications or results summaries: a systematic review and evidence-based recommendations.

You can find the full article here: <TBC>

You can access the full article for free.

Summary prepared by Dr Lauri Arnstein MBBS, Envision Pharma Group. Plain language services were funded by Envision Pharma Group. The other co-authors of the full article were involved in preparing this summary.
