# Supplementary material for: Patient involvement in preparing health research peer-reviewed publications or results summaries: a systematic review and evidence-based recommendations
Source: Res Involv Engagem. 2020 Jun 24;6:34. doi: 10.1186/s40900-020-00190-w (PMC7313171; doi:10.1186/s40900-020-00190-w)
Supplement: Supplementary file 2 — Additional file 2. Summary of search strategies. [file 40900_2020_190_MOESM2_ESM.docx]

**Patient involvement in preparing clinical research peer-reviewed publications or results summaries: A systematic review and recommendations**

**Supplementary material: Search strategies**

1. MEDLINE


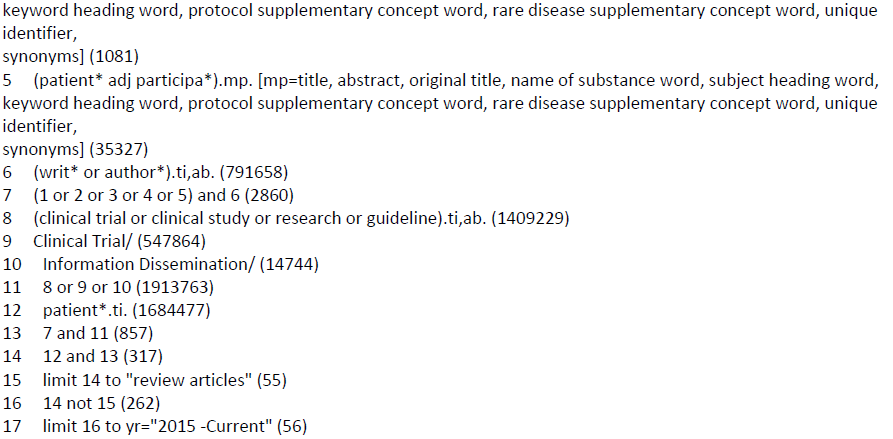


1. EMBASE

1. Cochrane Database of Systematic Reviews
