# Supplementary material for: Patient involvement in preparing health research peer-reviewed publications or results summaries: a systematic review and evidence-based recommendations
Source: Res Involv Engagem. 2020 Jun 24;6:34. doi: 10.1186/s40900-020-00190-w (PMC7313171; doi:10.1186/s40900-020-00190-w)
Supplement: Supplementary file 4 — Additional file 4. PAE tool – patient author version. [file 40900_2020_190_MOESM4_ESM.pdf]

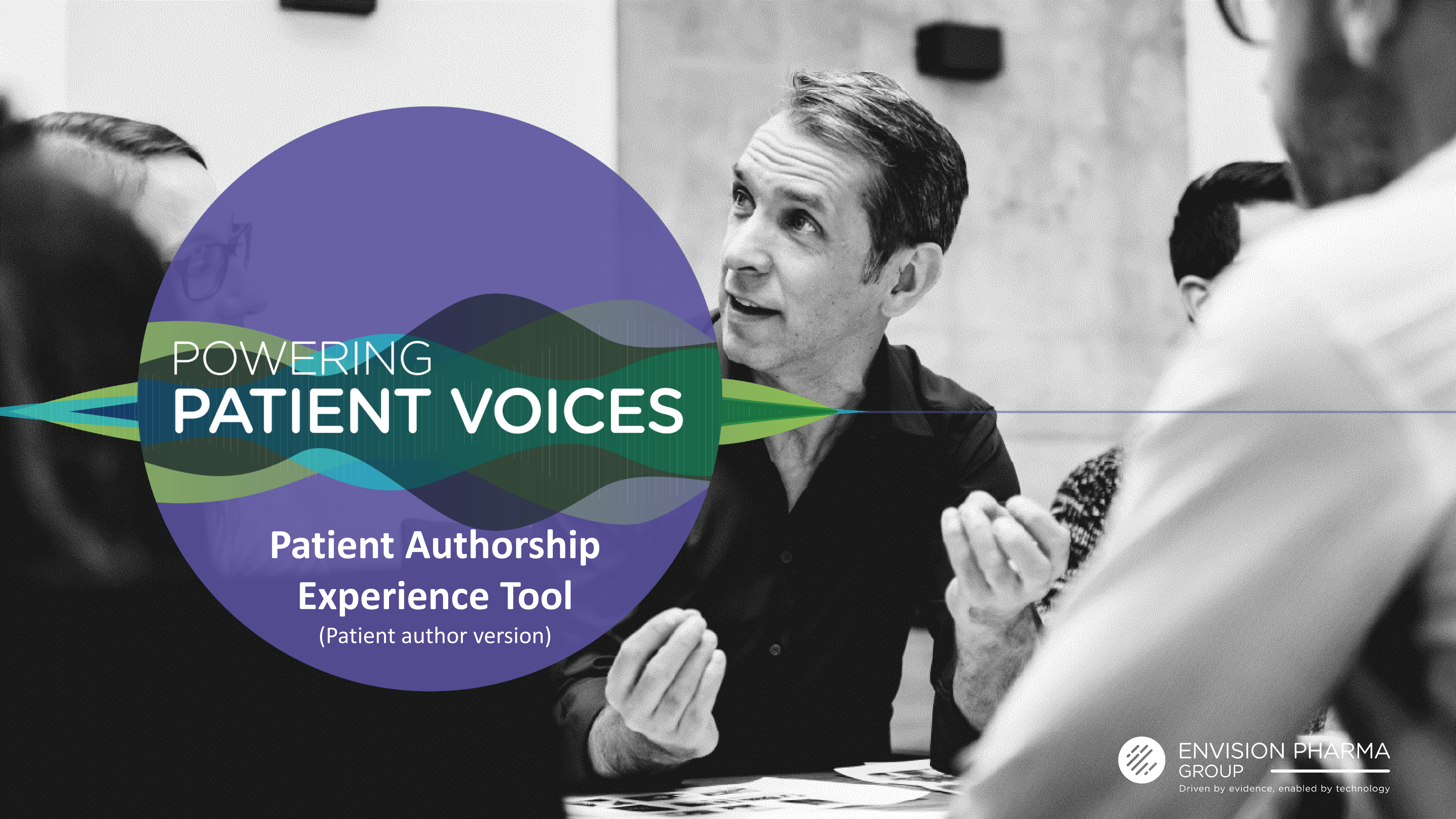

POWERING PATIENT VOICES

Patient Authorship Experience Tool

(Patient author version)

ENVISION PHARMA GROUP

Driven by evidence, enabled by technology

Patient authorship experience tool – a pilot test

- Currently, there is no validated 'quick and easy' tool to gain feedback directly from patient and non-patient authors on their experience when working together, as co-authors, on a publication .
- What is available?
  - The not-for-profit organization, Patient Focused Medicines Development, has developed a comprehensive assessment tool (17 pages; Patient Engagement Quality Guidance Tool [PEQG] to assess ongoing or completed patient engagement projects). This text-based tool is robust, but may take hours for authors to complete, and it is not focused on publications.
  - The Guidance for Reporting Involvement of Patients and the Public 2 (GRIPP2) checklist identifies what should be reported in publications and where. This checklist was primarily designed to facilitate reporting of PPI in publications; it was not designed to investigate the patient authorship experience.
- To help gain feedback directly, quickly, and easily on the patient authorship experience, we have developed a bipolar, 5-point, psychometric Likert scale*.
  - The tool has two versions
    1. For **patient authors** to complete
    2. For **non-patient authors** to complete
  - The tool is based on the PEQG, but focuses on the patient authorship experience. This tool is NOT intended to replace the PEQG tool or GRIPP2 guidelines.
  - The tool will be piloted with patient and non-patient authors as part of a co-creation process.

** To avoid left-to-right bias, the highest ratings have been positioned on the far right of the scale.*
Text descriptors have been included in each tick box to reinforce the direction of the scale.

Patient Authorship Experience Tool: Patient Author Version

Patients can be authors of peer-reviewed publications in scientific journals. However, patient authorship is still uncommon. We are keen to know how to make the authorship experience a positive one for **all** authors. This tool has been designed to gain feedback directly from patient authors about their authorship experience. Your feedback may help improve the authorship experience for other patients.

Name (desired, but optional):

Please circle the answer that best describes the number of peer-reviewed publications you authored before this publication

0	1-5	6-10	11 or more

Please read each statement, then circle the answer that describes how much you disagree or agree with the statement

Statement	Strongly Disagree	Disagree	Neutral	Agree	Strongly Agree
**1. Shared purpose**					
a. Along with my co-authors, I understood the aim of the publication	Strongly disagree	Disagree	Neutral	Agree	Strongly agree
b. My co-authors understood the importance of having a patient author	Strongly disagree	Disagree	Neutral	Agree	Strongly agree

Statement	Strongly Disagree	Disagree	Neutral	Agree	Strongly Agree
**2. Respect and accessibility**					
a. I was treated with respect during the development of the publication	Strongly disagree	Disagree	Neutral	Agree	Strongly agree
b. My ability to access and share information was taken into account (eg, ability to attend authors meetings, time to review documents)	Strongly disagree	Disagree	Neutral	Agree	Strongly agree
**3. Representativeness of stakeholders**					
a. The authorship group reflected the needs and interests of the end users of the publication	Strongly disagree	Disagree	Neutral	Agree	Strongly agree
b. I was able to represent patients who may be interested in being authors	Strongly disagree	Disagree	Neutral	Agree	Strongly agree
**4. Roles and responsibilities**					
a. I understood my roles and responsibilities of being an author, as outlined in the written authorship agreement form	Strongly disagree	Disagree	Neutral	Agree	Strongly agree
b. I was able to meet the four criteria for authorship, as outlined in the written authorship agreement form	Strongly disagree	Disagree	Neutral	Agree	Strongly agree
**5. Capacity and capability for engagement**					
a. I had sufficient time to make a useful contribution to the publication	Strongly disagree	Disagree	Neutral	Agree	Strongly agree
b. I had sufficient insights, from the patient perspective, to make a useful contribution to the publication	Strongly disagree	Disagree	Neutral	Agree	Strongly agree

Statement	Strongly Disagree	Disagree	Neutral	Agree	Strongly Agree
**6. Transparency in communication and documentation**					
a. Communication among authors was open and honest	Strongly disagree	Disagree	Neutral	Agree	Strongly agree
b. Documents (eg, outlines, drafts, minutes) were shared appropriately	Strongly disagree	Disagree	Neutral	Agree	Strongly agree
**7. Continuity and sustainability**					
a. I understood the main stages involved in preparing a publication	Strongly disagree	Disagree	Neutral	Agree	Strongly agree
b. Efforts were made to nurture relationships among the authorship group so that future projects may be considered	Strongly disagree	Disagree	Neutral	Agree	Strongly agree
**8. Results and outcomes**					
a. The research reported in this publication could have a positive impact on stakeholders (eg, patients, researchers)	Strongly disagree	Disagree	Neutral	Agree	Strongly agree
b. Efforts were made to learn from patients about their authorship experience	Strongly disagree	Disagree	Neutral	Agree	Strongly agree
**Final score**<br>To calculate the final score, add up the individual scores for each question (strongly disagree = 1; strongly agree = 5) and calculate a percentage out of a maximum score of 80					

Please add any other feedback here
